# Retraction: DMA, a Bisbenzimidazole, Offers Radioprotection by Promoting NFκB Transactivation through NIK/IKK in Human Glioma Cells

**DOI:** 10.1371/journal.pone.0234365

**Published:** 2020-06-03

**Authors:** The PLOS ONE Editors

Following the publication of this article [1], concerns were raised regarding Figs 5, 6, 7 and 9:

- Similarities were noted between 53 out of 68 western blot panels in Figs 5, 6 and 7. These included similarities between:
  ○ β-actin panels representing different conditions.
  ○ IKKα and IKKβ panels representing different conditions and/or different immunoblots.
  ○ P-IKKα and p-IKKβ panels representing different conditions and/or different immunoblots.
  ○ IκBα panels representing different conditions and/or different immunoblots.
  ○ IκBα panels and IKKβ panels representing different conditions.
- When adjusting colour levels of the panels listed below, it appears there are more than 6 lanes present in the panel, despite the figure legend indicating only 6 time points.
  ○ Fig 5: p-IKKα (control), 7 lanes detected, p-IKKβ (control), 7 lanes detected, and p-IKKβ (radiation), 8 lanes detected.
  ○ Fig 6: p-IKKα (control), 7 lanes detected and p-IKKβ (DMA), 8 lanes detected.
  ○ Fig 7, IKKα (DMA), 8 lanes detected.
- In Fig 9A, the DMA (50µM) + Radiation (8.5Gy), 3h panel appears similar to the DMA (50µM) + Radiation (8.5Gy), 6h panel. The authors indicate that the 3h panel was accidentally used to represent the 6h panel and have provided a replacement image for Fig 9A.

The authors were first approached by the journal regarding the concerns above in January 2020. The authors informed the journal they would provide the raw data underlying the figures of concern, but as of the time of the publication of this notice, the journal has not yet received the uncropped, original blots underlying Figs 5, 6, and 7. The authors indicated that the delay in providing the underlying data was due to institutional closures.

In light of the concerns affecting multiple figure panels that question the reliability of the reported results and conclusions, the *PLOS ONE* Editors retract this article.

VTa did not agree with the retraction and stands behind the published results. NK, AR, VTi, and RA either did not respond directly or could not be reached.
